# 

**Published:** 1897-06

**Authors:** 


					CONTENTS.
In Memoriam:
James Greig Smith...
103
Paroxysmal Tachycardia.
By P.
WATSON WILLIAMS, M.D. Lond. ...
122
Paralysis of the Respiratory Centre
from Intracranial Pressure.
Bv
J. Lacy Firth, M.S., M.D. Lond.,
RR.C.S. Eng
139
Two Cases of Abdominal Section.
By J. Raglan ihomas, m.JJ. Lond.,
M.R.C.S. Eng
145
A Case of Acute Miliary Tuberculosis.
By J. Michell Clarke, M.A., M.D.
Cantab., F.R.C.P
148-
A Case of Ruptured Tubal Pregnancy.
By W. H. C. Newnham, M.A.,
M.B. Cantab., M.R.C.S. Eng
152
Progress of the Medical Sciences:
Medicine.
George Parker, M.D.
134
Surgery.
Charles A. Morton. ...
161
Gynaecology and Obstetrics.
Walter C. Swayne, M.l>. ...
168
Reviews of Books
175
Notes on Preparations for the Sick
203
Bristol Medico-Chirurgical Society...
206
The Library of the Bristol Medico-
Chirurgical Society 214
Local Medical Notes
217
International Congress of Forensic
Medicine
218
Scraps
218

				

